# Older and Younger Adults Perform Similarly in an Iterated Trust Game

**DOI:** 10.3389/fpsyg.2021.747187

**Published:** 2021-10-12

**Authors:** Maïka Telga, Juan Lupiáñez

**Affiliations:** ^1^School of Management, University of St Andrews, St Andrews, United Kingdom; ^2^Experimental Psychology Department, University of Granada, Granada, Spain; ^3^Mind, Brain and Behavior Research Center (CIMCYC), University of Granada, Granada, Spain

**Keywords:** aging, individuation, age categories, gender categories, learning, trust

## Abstract

In social contexts, aging is typically associated with a greater reliance on heuristics, such as categorical information and stereotypes. The present research examines younger and older adults’ use of individuating and age-based categorical information when gauging whether or not to trust unfamiliar targets. In an adaptation of the iterated Trust Game, participants had to predict the cooperative tendencies of their partners to earn economic rewards in first encounters – in a context in which they knew nothing about their partners, and across repeated interactions – in a context in which they could learn the individual cooperative tendency of each partner. In line with previous research, we expected all participants to rely on stereotypes in first encounters, and progressively learn to disregard stereotypes to focus on individuating behavioral cues across repeated interactions. Moreover, we expected older participants to rely more on social categories than younger participants. Our results indicate that overall, both the elderly and the young adopted an individuating approach to predict the cooperative behaviors of their partners across trials. However, older adults more consistently relied on gender (but not age) stereotypes to make cooperation decisions at zero acquaintance. The impact of context, motivation, and relevance of categorical information in impression formation is discussed.

## Introduction

Aging is associated with cognitive impairments promoting the reliance on automatic processes in several domains of cognition, including social cognition. When forming impressions of others, older adults are more likely to rely on social heuristics, such as stereotypes and category-based judgments than younger adults. In the present research, we investigated older and younger adults’ reliance on individuating and age-based categorical information in first encounters, and when learning the individual cooperative behaviors of unfamiliar game partners in a Trust Game.

Being able to form accurate impressions of others is crucial to positive relations, and of particular relevance in the context of trust decisions. Because we typically trust others when we have a positive expectation about their future behaviors (Rousseau et al., 1998; Lewicki et al., 2006), the wisdom of our decisions depends on our ability to discriminate between trustworthy and untrustworthy interactions partners, and behave accordingly. Depending on several contextual and motivational variables, we may either focus on easily noticeable facial or social cues that help us to make quick decisions or focus instead on the individuating cues that allow us to understand our interactions partners as unique individuals.

In social interactions involving trust, perceivers are motivated to adopt an individuating approach, focusing on diagnostic individual behavioral information that is deemed valuable to predict future behaviors (Telga et al., 2018). When information about how others behaved in the past is available, it largely impacts trustworthiness judgments (Delgado et al., 2005; Shen et al., 2020). Similarly, when given the opportunity to interact multiple times with unfamiliar partners, perceivers typically observe and monitor the behavior of their partners, and base their trust decisions on the behavioral information acquired through experience in a reciprocal fashion, showing more trust with trustworthy individuals, and less trust with untrustworthy individuals (Axelrod and Hamilton, 1981; King-Casas et al., 2005; Alós-Ferrer and Farolfi, 2019; Bailey et al., 2019).

However, integrating the information acquired across several interactions to make sense of others is cognitively demanding, and people do not always have the relevant information, attentional resources or motivation to involve in such individuating processes. Prominent models of person perception suggest that in these contexts, perceivers may rely on social heuristics to decide how to act. In particular, they largely rely on the information related to targets’ social categories to inform their judgments and guide their behavior (Fiske and Neuberg, 1990; Macrae and Bodenhausen, 2000; Kawakami et al., 2017).

Although research has largely demonstrated that younger, middle-aged, and older adults are likely to use social categories to form impressions of others, the reliance on categorical versus individuating information varies across life span. In fact, aging is associated with cognitive losses related to memory, speed processing, reasoning, or executive functions (Salthouse, 2010; Reuter-Lorenz and Park, 2014), and these functions are involved in many daily activities, including social interactions (von Hippel, 2007). In this context, cognitive impairments affect several social abilities, such as behavioral restraint, emotion recognition, or perspective-taking (Moran et al., 2012; Kalokerinos et al., 2017). Importantly, age-related deficits differentially impact controlled and automatic processes: while automatic processes are rather well preserved across life span, controlled and inhibitory processes are more deficient in older compared to younger adults (von Hippel and Henry, 2012). Research has demonstrated that older adults’ impairments in inhibitory processes lead them to rely more on the preserved automatic processes, resulting in a larger use of heuristics in contexts related to learning (Price and Murray, 2012), language (Kim et al., 2005), financial decision making (Chen and Sun, 2003), memory (Fine et al., 2018), and social cognition. In particular, when it comes to impressions formation, older adults are more likely to rely on heuristics related to social categories than younger adults (Gonsalkorale et al., 2009; Radvansky et al., 2010).

Social categories, such as age, gender, or ethnicity, are automatically and effortlessly extracted at early stages of face processing (Mouchetant-Rostaing et al., 2000; Hugenberg and Wilson, 2013). Once a person has been categorized, the set of knowledge and beliefs held about his or her group shapes downstream our evaluation and assumptions about this person (Freeman and Ambady, 2011; Stolier and Freeman, 2016). For instance, Montepare and Zebrowitz (1998) showed that on the social dimension of age, features related to a baby-face, such as round cheek, large eyes, and high eyebrows, trigger age-related traits, in such a way that more typical baby-faces are associated with traits, such as submission or dependence. Such categorical information also impacts trustworthiness judgments. In fact, perceivers readily activate stereotypes associating older adults with more trustworthiness and cooperation than younger adults (Cuddy et al., 2005; Schniter and Shields, 2014), which may impact cooperation decisions. Therefore, stereotypes are rules of thumb that perceivers may rely on to make quick judgments about unfamiliar interaction partners. Because the inhibition of automatically activated stereotypical judgments involves controlled processes (Gilbert and Hixon, 1991), older adults are typically less able to inhibit the automatic activation of stereotypes, resulting in more stereotypic decisions and prejudiced judgments (Gonsalkorale et al., 2009; Radvansky et al., 2010). In the present research, we therefore expected older adults to rely on stereotypes when making inferences about interaction partners at zero acquaintance.

Categorizing others not only triggers stereotypical judgments but may also activate biases related to self-perception. When organizing the social world in terms of categories, we necessarily realize that there are social groups to which we belong (e.g., ingroup) and groups to which we do not (i.e., outgroup; Ellemers, 2012; Ellemers and Haslam, 2012). These self-categorization processes may impact our perception of and interactions with others in several different ways (Turner et al., 1987). For instance, outgroup members are typically perceived as more similar to each other, while ingroup members appear more heterogeneous (Tajfel and Wilkes, 1963). Consequently, outgroup members are often perceived and evaluated along with the information related to their category membership, while ingroup members are individuated and evaluated along with their unique characteristics (Judd and Park, 1988; Freeman et al., 2010).

Importantly, this outgroup homogeneity effect is also observed in the context of trust decisions and may persist across repeated interactions. For instance, Telga et al. (2018) used an iterated Trust Game to explore participants’ learning of the cooperative behaviors of ingroup and outgroup members. Within each social group, a small proportion of individuals (i.e., inconsistent partners) showed a pattern of cooperation opposite to the rest of their group. Their results suggested that categorical information had a higher impact on the learning of outgroup as compared to ingroup partners’ cooperation tendency. In fact, participants learned to a lesser extent about inconsistent outgroup members, as compared to inconsistent ingroup members. The present research aimed to extend previous findings on the outgroup homogeneity effect in trust decisions. In particular, we explored whether younger adults also show an outgroup homogeneity effect with age (instead of gender or ethnicity) categories. Moreover, going beyond previous studies testing trust decisions in a one-shot paradigm (Bailey et al., 2015), we examined older adults’ reliance on categorical information across repeated interactions, in a context in which they had to keep in mind the cooperative tendencies of eight different partners across time. With this paradigm, we investigated whether older adults are prone to show an outgroup homogeneity effect, categorizing more ingroup compared to outgroup members, or alternatively, mostly rely on categorical information, categorizing both ingroup and outgroup members.

Categorical information may also be used to guide our perception and decisions by applying newly learned categorical information to novel group members with whom we lack experience (Crawford et al., 2002; Ranganath and Nosek, 2008). Specifically, if we learn to associate a specific social group to a particular behavior, we may use this information to cooperate with newly encountered members of this group (Cañadas et al., 2013). In cooperation decisions, if participants learned across trials that one group is highly cooperative while the other one is not, they may be more likely to cooperate with novel members of the former group, and less likely to cooperate with novel members of the latter (Vermue et al., 2019). The present research explored whether this member-to-member categorization process is more prominent among older as compared to younger adults.

The cognitive impairments associated with aging not only promote reliance on social heuristics in the form of stereotypes and category-based judgments, but also impact how older adults engage in social interactions. For instance, Labouvie-Vief (2003) argued that as we grow older, we may adaptatively shift our focus of attention from negative to less cognitively demanding positive information. Consistently, in the domain of trust, older adults show an attentional bias favoring positive over negative information, especially when presented with negative indicators of trustworthiness (see Bailey and Leon, 2019, for a meta-analysis). This converging evidence suggests that in social interactions involving trust, older adults may be less sensitive to negative information related to untrustworthiness than younger adults. Additionally, older adults may disattend negative untrustworthy stimuli not only as an adaptative strategy to preserve impaired cognitive resources, but also because it would benefit them at the emotional and social levels. From this perspective, Carstensen et al. (1999) suggested that facing decreasing future perspectives, older adults may be more motivated to focus on emotionally satisfying social interactions. In fact, trust increases with age, and higher levels of trust have been related to the elderly’s emotional wellbeing (Li and Fung, 2013; Poulin and Haase, 2015). Finally, from a slightly different perspective, Charness and Villeval (2009) proposed that by showing more trust and cooperation, older adults may try to adopt a role model for younger adults, “teaching” them the benefits of reciprocal exchanges. Altogether, these theories suggest that older adults may overall demonstrate more trust than younger adults.

In sum, the decreasing of cognitive functions with age may impact cooperation and trust decisions in different ways. Older adults may rely to a greater extent on categorical information when forming impressions of others and assessing their trustworthiness. Also, older adults may be less sensitive to negative feedback of untrustworthiness, and overall show larger cooperation rates than their younger counterparts. These hypotheses are explored in an adaptation of the iterated Trust Game (Berg et al., 1995; Telga et al., 2018), by comparing older and younger adults’ cooperation decisions in first interactions (baseline phase and transfer phases) and when learning the trustworthiness tendency of unfamiliar young and old partners (learning phase).

First, we explored spontaneous first impressions about older and younger partners by creating a context where neither individual behavior nor social categories were predictive of partners’ behaviors. Specifically, in a baseline phase, participants were presented with four older and four younger partners, all of them cooperating on half of the trials, and not cooperating on the other half. We expected all participants to rely on age-related stereotypes associating older adults to more trustworthiness, and therefore to cooperate more with older than younger partners (Hypothesis 1).

Second, we examined the impact of categorical (partners’ group membership) and individual (partners’ individual behaviors) information on cooperation decisions across multiple interactions. In a subsequent learning phase, the same eight partners presented in the baseline were again presented but now displaying specific cooperation tendencies. Specifically, the partners belonging to one of the categories (either young or old) were cooperative and cooperated with the participants on most trials, whereas those belonging to the other category were noncooperative and did not cooperate with participants on most trials. While within each age group, most partners displayed the same cooperative behaviors (either cooperative or noncooperative), a small proportion of inconsistent partners displayed a cooperative tendency opposite to the group behavior. With this paradigm, individual behaviors are the most valuable predictors of partners’ future behaviors. Although relying on categorical information should lead participants to predict the cooperative behaviors of most of their partners (i.e., consistent partners), they would be less successful in predicting the behaviors of inconsistent partners, and therefore, would be overall less accurate than if they relied on individuating information.

We expected the pattern of results in the learning phase to echo previous findings observed with gender and ethnic categories. In particular, Telga et al. (2018) found that younger participants’ learning about gender and ethnic outgroups is impacted by an outgroup homogeneity effect. In the present research, we also expected younger participants to show an outgroup homogeneity effect with age categories. Specifically, we anticipated that younger participants would learn to a similar extent the cooperative tendency of consistent and inconsistent ingroup partners (i.e., young partners) but would learn more about the cooperative tendencies of consistent compared to inconsistent outgroup partners (i.e., older partners) across repeated interactions (Hypothesis 2a). As for older participants, we explored three hypotheses that we considered substantially supported by the existing literature, and therefore, equally likely. First, that older participants also show an outgroup homogeneity effect (Hypothesis 2b), and therefore learn less about the cooperative behaviors of inconsistent outgroup (i.e., younger partners) than inconsistent ingroup (i.e., older partners). Second, that when assessing the cooperative tendencies of unfamiliar people, older adults primarily rely on categorical information when interacting with both ingroup and outgroup partners (Hypothesis 2c). This effect should be reflected in a poorer learning of the cooperation tendencies of inconsistent compared to consistent partners. Third, that older adults show an overall higher tendency to cooperate than younger adults (Hypothesis 2d).

Finally, in a transfer phase, participants were presented with four new older and four new younger partners with whom they had no prior experience, all of them being cooperative on half of the trials. In this phase, we explored whether the categorical associations learned in the learning phase would impact cooperation decisions with novel older and younger adults, that is, whether learning to associate one age group to more cooperation would lead to more cooperation with novel members of this group. The hypotheses and procedure of this experiment were pre-registered on Open Science Framework.^1^

## Materials and Methods

### Participants

Before data collection, we decided to run a minimum of 40 participants in each group, as in previous research using this paradigm (Telga et al., 2018). Accordingly, 41 older (24 men, mean age: 65.36, range: 60–86) and 41 younger (12 men, mean age: 21.78, range: 18–27) volunteers participated in the study in exchange for a financial compensation proportional to their performance in the task (€5.40 on average). A sensitivity analysis using G*Power 3.1 (Faul et al., 2007) found that this sample could detect an effect of $\eta_{p}^{2}$=0.09 (power=0.80, *α*=0.05) for the predicted main effect of Partner Age in the baseline phase. All participants reported normal or corrected to normal vision. Written informed consent was obtained from all participants and the study was part of a larger project approved by the local university ethical committee (175/CEIH/2017).

### Procedure

Upon arrival to the lab, participants provided consent and were led to individual cubicles. Older participants were individually administered the Mini-Mental State Examination (MMSE, Folstein et al., 1983). All participants were then provided with visual and verbal instructions about the trust game. Participants were informed that they would have to decide whether or not to cooperate with unfamiliar people in several rounds. Each round started with “€1” displayed for 200ms indicating that participants virtually received €1. After a 200-ms fixation point, the photograph of the partner of this round appeared during 1,000ms and participants were asked to make their decision by pressing the “1” key cooperate or the “0” to keep the euro and therefore, not to cooperate. Keeping the €1 led to the end of the round. Alternatively, if participants cooperated, their partners would receive €5 and in turn decide whether or not to reciprocate by sending back €2.50 or keeping the €5 to themselves, leaving the participant with nothing. After participants made their decision or after 1,500ms, a 500-ms fixation cross was displayed followed by the partner’s decision and the final outcomes in a single screen during 1,500ms. A 1,000ms inter-trial black screen ended each round. Participants were informed of the payoffs structure and were encouraged to maximize their benefits.

**Figure 1:**
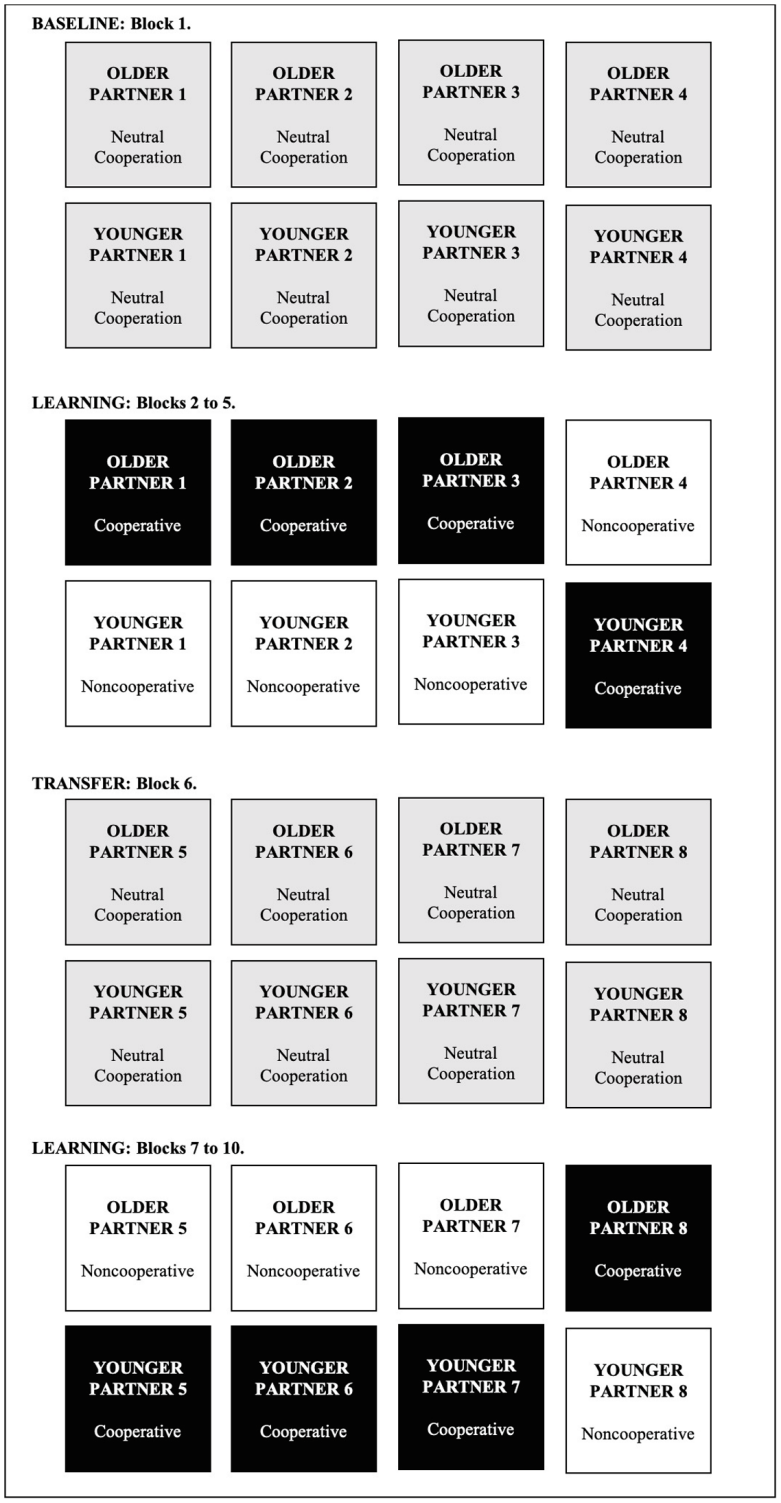
Example of the procedure employed for the trust game. Consistent partners are represented in white and inconsistent partners are represented in black. In the baseline and transfer phases, partners are presented for the first time and their cooperative behavior is not manipulated.

As shown in Figure 1, the Trust Game started with a baseline phase (Block 1). In this phase, participants were presented with four older and four younger partners (two men and two women in each age group). All partners cooperated on half of the trials and did not cooperate on the other half, thus making their individual cooperative behaviors uninformative of whether or not to cooperate. Moreover, partners’ cooperation was independent of their age category, thus making social category membership uninformative of whether or not to cooperate as well. Each partner was presented eight times for a total of 64 trials. The order of presentation of the trials was randomized independently for each participant.

Next, in the subsequent first learning phase (Blocks 2 to 5), partners’ social category and individual behaviors became meaningful to predict their future responses. Specifically, each age group was associated with a particular cooperative tendency, either cooperative or noncooperative. In the cooperative group (e.g., older partners), three partners cooperated on 75% of the trials while in the noncooperative group (e.g., younger partners), three partners cooperated only on 25% of the trials. Moreover, in each age group, the fourth partner was inconsistent with respect to the rest of their group. That is, in the cooperative group, one partner (e.g., one old partner) cooperated only on 25% of the trials. Conversely, in the noncooperative group, one partner (e.g., one young partner) cooperated on 75% of the trials. Importantly, inconsistent partners displayed a cooperative tendency inconsistent with the group behavior but, at the individual level, always displayed the same cooperation tendency for the entire task. With this paradigm, if participants rely on categorical information to make their cooperation decision, they should focus on the group behavior and cooperate to a similar extent with all partners of the same age, independently of whether they behavior is consistent or inconsistent with the group behavior. This should be reflected in a main effect of the group behavior variable, not qualified by consistency. If, in contrast, participants rely on individuating behavioral information, they should show opposite patterns of cooperation for consistent and inconsistent partners within the same age group, cooperating with each partner according to their individual behavior. This should be reflected in a Group Behavior×Consistency interaction showing more cooperation with consistent partners belonging to the cooperative group and inconsistent partners belonging to the noncooperative group, and less cooperation with consistent partners belonging to the noncooperative group and inconsistent partners belonging to the cooperative group. Each partner was presented 32 times, resulting in 256 trials divided into four blocks of 64 trials. Within each block, each partner was presented eight times. Cooperative partners cooperated on 75% of the trials (six out of eight trials) and did not cooperate on 25% of the trials (two out of eight trials). Noncooperative partners cooperated on only 25% of the trials (two out of eight trials) and did not cooperate on 75% of trials (six out of eight trials). The order of presentation of the trials was randomized within each block of 64 trials, independently for each block and for each participant. The faces used to represent the two inconsistent partners were counterbalanced such that across participants, all faces were associated with the inconsistent condition.

Next, participants played the transfer phase with four new older and four new younger partners (two men and two women in each group). In this phase, all partners cooperated on half of the trials and did not cooperate on the other half of the trials. Therefore, neither age groups nor individual behavior was informative of partners’ likelihood to cooperate. This phase allowed us to examine whether participants used the categorical associations established in the learning phase to categorize new partners. Specifically, in this context, reliance on categorical information should be reflected in more cooperation with partners belonging to the age group that was cooperative in the learning phase, and less cooperation with new partners from the age group that was noncooperative in the learning phase. Alternatively, a greater reliance on individuating information should be reflected in cooperation rates independent of age groups. Each partner was presented eight times for a total of 64 trials, and the order of presentation of the trials was randomized independently for each participant.

Finally, in a second learning phase (Blocks 7 to 10), we counterbalanced the associations established between age groups and group behaviors. The same partners used for the transfer phase were again presented, now displaying specific patterns of cooperation. Similarly to Blocks 2 to 5, the two age groups displayed opposite patterns of behaviors and within each group, 25% of the partners displayed a cooperative tendency opposite to the group behavior. Importantly, in this second learning phase, we counterbalanced the associations established between age groups and group behavior (e.g., older partners cooperative and younger partners noncooperative). Specifically, if older partners were cooperative and younger partners were noncooperative in Block 2 to 5, older partners would be noncooperative and younger partners would be cooperative in Blocks 7 to 10. This procedure allowed us to have a full within-participants design regarding the variables of interest. Whether younger or older partners were cooperative in the first (Blocks 2 to 5) or second (Block 7 to 10), learning phase was counterbalanced across participants.

### Stimuli and Materials

Computers equipped with a 24-inch monitor and E-Studio 2.0 software (Schneider et al., 2002) were used for stimuli presentation and data acquisition. The game partners were represented by 16 photographs taken from the UT Dallas Face Database (Minear and Park, 2004). All faces were presented against a gray background with a neutral emotional expression. Older partners were between 61 and 68years while younger partners were between 20 and 25years. All partners were white and within each group of age, half of the partners were men and the other half were women.

### Design and Analyses

For all analyses, participants’ cooperation rate, that is, the proportion of trials on which participants chose to cooperate, was analyzed as a dependent variable. For instance, if in a particular condition a participant cooperates on 5 out of 100 trials (and, therefore, does not cooperated on 95 out of 100 trials), their cooperation rate in that condition would be 0.05.

In the baseline phase, cooperation rates were analyzed as a function of partner age (whether the partner on a given trial is elderly or young) and participant age (whether the participant is elderly or young), resulting in a 2 (Partner Age: older vs. younger)×2 (Participant Age: older vs. younger) mixed design, with the latter variable as a between-participants factor. In this phase, we expected all participants to cooperate more with older than younger partners (Hypothesis 1). This should be reflected in a main effect of Partner Age indicating higher cooperation rates with older than younger partners.

In the learning phases, cooperation rates were analyzed as a function of partner group (whether the partner on a given trial is from the participant’s ingroup or outgroup), group behavior (whether the partner on a given trial belongs to the cooperative or noncooperative group), consistency (whether the partner on a given trial behaves in a way that is consistent or inconsistent with the group behavior), and block (from what block is a particular trial taken) as within-participants variables, and participant age (older vs. younger) as a between-participants factor. Note that because the variable cooperative group (i.e., whether ingroup or outgroup partners are cooperative) is counterbalanced across the two phases of learning (Block 2 to 5 and Block 7 to 10), it is necessary to combine these two learning phases^2^ to verify the hypotheses related to an outgroup homogeneity effect (Hypotheses 2a and 2b) for a full within-subjects design regarding the variables of interest.

In the learning phase, an effect of the block variable would reflect how participants’ expectations about their partners’ cooperative behaviors evolve as they acquire information about these partners. Learning is typically reflected in an increase in cooperation with cooperative partners across blocks and a decrease in cooperation with noncooperative partners across blocks. Whether this block variable interacts with group behavior or consistency is informative of whether participants learned to attend to categorical or individual behavioral information.

Specifically, a Group Behavior×Block interaction, not qualified by Consistency, would indicate that when gauging whether or not to cooperate with a given partner, participants rely exclusively on categorical information and do not notice inconsistent individuals at all. Based on previous research using the trust game showing that the impact of categorical information is subtler (Telga et al., 2018), we did not anticipate such an effect.

We, however, expected a significant Group Behavior×Consistency×Block. In that case, the analysis of participants’ pattern of cooperation with consistent partners on the one hand, and inconsistent partners, on the other hand, would be informative of the reliance on either categorical or individual cues.

In particular, the analysis of cooperation with consistent partners is informative of participants’ capacity to infer the cooperative behavior of their partners when consistent with categorical information. In this condition, we typically expect participants to increase their cooperation with consistent partners belonging to the cooperative group and decrease their cooperation with partners belonging to the noncooperative group, which should be reflected in a significant Group Behavior×Block interaction.

The analysis of cooperation with inconsistent partners indicates to what extent were participants able to detect, discriminate, and accurately adjust their cooperation with these inconsistent individuals who displayed a cooperation tendency opposite to the group behavior. This type of adjustment can only be achieved if participants focused on the individual behavioral tendency of their partners, regardless of the social category they belong to. Such individuating approach should be reflected in participants responding to their partners’ individual behavior. Because inconsistent individual displayed a pattern of cooperation opposite to the group behavior, this means increasing their cooperation with inconsistent individuals belonging to the noncooperative group and decreasing their cooperation with inconsistent individuals belonging to the cooperative group, which should also be reflected in a significant Group Behavior×Block interaction. If, alternatively, the Group Behavior×Block interaction is not significant in the inconsistent partners condition, this would mean that participants failed to learn the cooperative behaviors of partners whose cooperative behaviors differed from the group’s.

In sum, a greater reliance on individual behavioral cues should be reflected in significant Group Behavior×Block interaction in both consistent and inconsistent partners conditions, and a greater reliance on categorical cues should be reflected in a significant Group Behavior×Block interaction in the consistent condition, but the same interaction not being significant in the inconsistent partners condition.

Therefore, if older participants rely on categorical information to a greater extent than younger participants (Hypothesis 2c), the aforementioned Group Behavior×Consistency×Block interaction should be qualified by Participant Age, resulting in a significant four-way Participant Age×Group Behavior×Consistency×Block interaction. If both younger (Hypothesis 2a) and older (Hypothesis 2b) participants show an outgroup homogeneity effect, the same interaction should be qualified by Partner Group, resulting in a significant four-way Partner Group×Group Behavior×Consistency×Block interaction. If the hypothesis that older participants are more cooperative than younger participants is verified (Hypothesis 2d), we should observe a main effect of Participant Age showing higher cooperation rates for older than for younger participants.

Finally, in the transfer phase, we analyzed cooperation rates as a function of partner age, participant age, and the information learned in the first learning phase (i.e., whether older adults were mostly cooperative in the first learning, or younger adults were mostly cooperative in the first learning phase), resulting in a 2 (Partner Age: older vs. younger)×2 (Learned Cooperative Group: older vs. younger partners)×2 (Participant Age: older vs. younger) mixed design with the first variables as within-participants factors. If participants solely rely on age-related stereotypes in the transfer phase, we should observe a main effect of Partner Age with more cooperation with older than younger partners. If participants relied on the information learned in the first learning phase, they should cooperate more with partners from the age group associated with cooperative behaviors in the first learning phase (i.e., more cooperation with older partners and less cooperation with younger partners when older partners were cooperative in the first learning phase. Alternatively, more cooperation with younger partners and less cooperation with older partners when younger partners were cooperative in the first learning phase). This should be reflected in a significant Partner Age×Learned Cooperative Group interaction. Any difference between the two participants age group should be reflected in an interaction with the Participant Age variable.

## Results

Data from one older participant were excluded from the analyses for abandoning the experiment before its end, leaving in 41 younger adults and 40 older adults for the analyses. All the remaining participants were included in the analyses, as all older participants passed our inclusion criterion, obtaining a score higher than 27 (mean: 29.08, range: 28–30) at the MMSE. The rejection criterion was set at 0.05 for all statistical tests and 95% confidence intervals on $\eta_{p}^{2}$ were computed following Nelson (2016).

### Baseline

To test the hypotheses that all participants would cooperate more with older than younger partners (Hypothesis 1), we introduced cooperation rates in the baseline phase (Block 1) in a mixed-design ANOVA with partner age (older vs. younger) as a within-participants variable and participant age (older vs. younger) as a between-participants factor. The expected main effect of partner age was not significant *F*(1, 79)=1.27, *p*=0.263, $\eta_{p}^{2}$=0.02, 95% CI=[0.00, 0.09], as participants showed similar cooperation rates with older (*M*=0.64, *SD*=0.19) and younger (*M*=0.66, *SD*=0.16) partners. Hypothesis 1 was, therefore, not supported. Moreover, neither the main effect of participant age, *F*(1, 79)=1.14, *p*=0.290, $\eta_{p}^{2}$=0.01, 95% CI=[0.00, 0.08], nor the Partner Age×Participant Age interaction, *F*(1, 79)=0.69, *p*=0.410, $\eta_{p}^{2}$=0.01, 95% CI=[0.00, 0.07] was significant, suggesting that older and younger participants approached their partners in a similar way in first encounters.

### Learning Phase

To verify whether participants’ learning about the cooperative behaviors of their partners was impacted by an outgroup homogeneity effect (Hypotheses 2a and 2b), we conducted a mixed-design ANOVA on cooperation rates in the learning phase with partner group (ingroup vs. outgroup), group behavior (cooperative vs. noncooperative), consistency (consistent vs. inconsistent), and block (II, III, IV, V) as within-participants variables, and participant age as a between-group factor. The Group Behavior×Consistency×Block interaction was significant, *F*(3, 237)=34.88, *p*<0.001, $\eta_{p}^{2}$ =0.31, 95% CI=[0.22, 0.37], superseding the interpretation of any other principal effect and indicating that overall, participants adopted an individuating approach when deciding whether or not to cooperate with their partners. The Partner Group×Group Behavior×Consistency×Block interaction that would indicate an outgroup homogeneity effect was not significant, *F*(3, 237)=1.43, *p*=0.236, $\eta_{p}^{2}$=0.02, 95% CI=[0.00, 0.04], indicating that Hypotheses 2a and 2b were not supported. The Participant Age×Group Behavior×Consistency×Block that would indicate different learning patterns between older and younger participants was not significant either, *F*(3, 237)=1.16, *p*=0.325, $\eta_{p}^{2}$=0.01, 95% CI=[0.00, 0.04], indicating that Hypothesis 2c was not supported. Finally, the main effect of participants’ age that would indicate that older participants trust more than younger participants was not significant, *F*(1,79)<0.01, *p*=0.997, $\eta_{p}^{2}$<0.01, indicating that Hypothesis 2d was not supported. To decompose the significant Group Behavior×Consistency×Block interaction, we first analyzed cooperation with consistent partners (i.e., those who behaved consistently with the group behavior) and next turned to inconsistent individuals (i.e., those who displayed a pattern of cooperation opposite to the group behavior).

**Figure 2:**
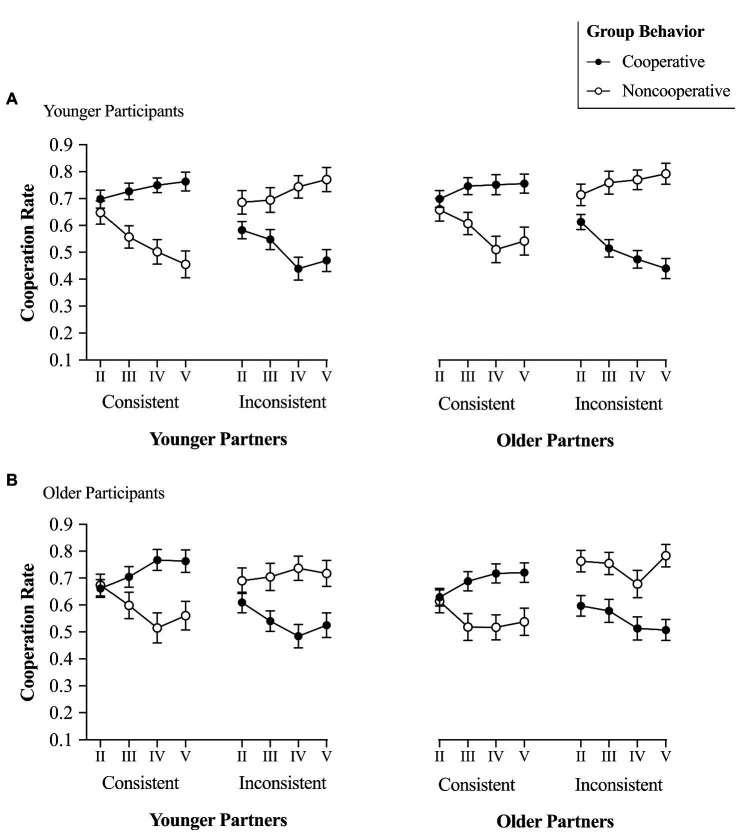
Cooperation rates in the learning phase as function of partners’ consistency (consistent vs. inconsistent) and group behavior (cooperative vs. noncooperative) for **(A)** younger and **(B)** older participants. Error bars represent the error standard of the mean.

In the consistent partners condition, the Group Behavior×Block was significant, *F*(3, 240)=41.66, *p*<0.001, $\eta_{p}^{2}$ =0.34, 95% CI=[0.26, 0.41]. In fact, when partners were cooperative, participants linearly increased their cooperation from Block II (*M*=0.67, *SD*=0.17) to Block V (*M*=0.75, *SD*=0.21), *F*(1, 80)=30.15, *p*<0.001, $\eta_{p}^{2}$ =0.27, 95% CI=[0.15, 0.39]. The quadratic component was also significant, *F*(1, 80)=6.67, *p*=0.012, $\eta_{p}^{2}$=0.08, 95% CI=[0.01, 0.18], indicating that this increase reached an asymptote as shown in Figure 2. In contrast, when partners were noncooperative, participants linearly decreased their cooperation from Block II (*M*=0.60, *SD*=0.18) to Block V (*M*=0.48, *SD*=0.24), *F*(1, 80)=43.63, *p*<0.001, $\eta_{p}^{2}$=0.36, 95% CI=[0.21, 0.46], until reaching an asymptote, quadratic component, *F*(1, 80)=9.35, *p*=0.003, $\eta_{p}^{2}$=0.11, 95% CI=[0.02, 0.22].

In the inconsistent partners condition, that is, when partners displayed cooperative behaviors opposite to the group behavior, the Group Behavior×Block interaction was also significant, *F*(3, 237)=13.05, *p*<0.001, $\eta_{p}^{2}$=0.14, 95% CI=[0.07, 0.20], showing that participants accurately adjusted their cooperation with inconsistent partners. Specifically, they linearly increased their cooperation with inconsistent partners belonging to the noncooperative group from Block II (*M*=0.71, *SD*=0.21) to Block V (*M*=0.76, *SD*=0.21), *F*(1, 80)=4.55, *p*=0.036, $\eta_{p}^{2}$= 0.05, 95% CI=[0.00, 0.15], and linearly decreased their cooperation with inconsistent partners belonging to a cooperative group from Block II (*M*=0.65, *SD*=0.22) to Block V (*M*=0.52, *SD*=0.28), *F*(1, 80)=25.18, *p*<0.001, $\eta_{p}^{2}$=0.24, 95% CI=[0.11, 0.36], until reaching an asymptote, quadratic component, *F*(1, 80)=8.62, *p*=0.004, $\eta_{p}^{2}$=0.10, 95% CI=[0.01, 0.21].

Following a reviewer’s suggestion, we also analyzed the two learning phases separately and observed that in the first learning phase (Blocks 2 to 5), older and younger adults learned to the same extent about consistent and inconsistent individuals. In fact, the Group Behavior×Consistency×Block interaction was significant, *F*(3, 231)=26.88, *p*<0.001, $\eta_{p}^{2}$=0.26, 95% CI [0.18, 0.33], and not qualified by Participant Age, *F*(3, 231)=0.19, *p*=0.901, $\eta_{p}^{2}$<0.01, 95% CI [0.00, 0.01]. In the second learning phase (Blocks 7 to 10), however, older and younger participants’ performance seemed to differ, as the Participant Age×Group Behavior×Consistency×Block was not significant but close, *F*(3, 231)=2.48, *p*=0.062, $\eta_{p}^{2}$=0.03, 95% CI [0.00, 0.07], suggesting that in this second learning phase, both older and younger adults discriminated between consistent and inconsistent individuals, but for older participants, the general tendency to cooperate more with individually cooperative individuals and less with individually noncooperative individuals did not increase across blocks. The full breakdown of these analyses is provided in Supplementary Materials.

### Transfer

To explore the hypothesis that participants would use the information acquired in the learning phase to cooperate with novel older and younger partner, we conducted a mixed-design ANOVA on cooperation rates in the transfer phase with partner age (older vs. younger) as a within-participant variable and learned cooperative group (older vs. younger partners) and participant age (older vs. younger) as between-group factors. Neither the main effect of participant age, *F*(1, 77)=0.93, *p*=0.339, $\eta_{p}^{2}$=0.01, 95% CI=[0.00, 0.08], nor the main effect of partner age, *F*(1, 77)=0.65, *p*=0.422, $\eta_{p}^{2}$<0.01, 95% CI=[0.00, 0.07] was significant. The Cooperative Group×Partner Age interaction was not significant either, *F*(1, 77)=3.16, *p*=0.080, $\eta_{p}^{2}$=0.04, 95% CI=[0.00, 0.13]. No other interaction was significant, being the larger F for the Participant Age×Cooperative Group×Partner Age interaction, *F*(1, 77)=1.19, *p*=0.278, $\eta_{p}^{2}$=0.02, 95% CI=[0.00, 0.09].

### Exploratory Analyses

Given that participants did not seem to use age groups either in the baseline phase or in the transfer phase, we decided to explore whether a different social dimension for categorization may have overshadowed age. Notably, given that within each age group, half of the partners were female, we analyzed whether participants used gender instead of age to categorize their partners in the baseline and the transfer phases. Therefore, we repeated the same analyses as described above but introduced partner gender as a within-participants factor.

Specifically, to analyze a potential impact of partners’ gender on participants’ decisions in the baseline, cooperation rates were subjected to a 2 (Partner Age: older vs. younger)×2(Partner Gender: female vs. male)×2(Participant Age: older vs. younger) mixed-design ANOVA. We observed a Partner Gender×Participant Age interaction, *F*(1, 79)=11.32, *p*=0.001, $\eta_{p}^{2}$=0.13, 95% CI=[0.03, 0.24]. While younger participants seemed to cooperate slightly more with female (*M*=0.67, *SD*=0.15) than with male (*M*=0.65, *SD*=0.16) partners, this effect was not significant, *F*(1, 40)=0.35, *p*=0.560, $\eta_{p}^{2}$<0.01, 95% CI=[0.00, 0.10]. However, older participants significantly cooperated more with female (*M*=0.72, *SD*=0.15) than with male (*M*=0.54, *SD*=0.24) partners, *F*(1, 39)=19.43, *p*<0.001, $\eta_{p}^{2}$=0.33, 95% CI=[0.14, 0.49].

Next, to explore whether gender impacted participants’ decision in the transfer phase, we conducted a mixed-design ANOVA on cooperation rates with partner age and partner gender as within-participants variables, and group behavior and participant age as between-participants factors. The only significant effect was the main effect of partner gender, *F*(1, 79)=7.24, *p*=0.009, $\eta_{p}^{2}$=0.08, 95% CI=[0.01, 0.19], indicating that participants cooperated more with female (*M*=0.70, *SD*=0.16) than with male (*M*=0.64, *SD*=0.19) partners.

## Discussion

The present research explores the impact of categorical information related to age on older and younger adults’ cooperation decisions in a Trust Game. Specifically, we analyzed spontaneous demonstrations of trust in first encounters (baseline and transfer phases) as well as learning of the cooperative behaviors of unfamiliar partners across repeated interactions (learning phases).

Across repeated interactions, the individuating information was prioritized over heuristics as both older and younger participants noticed and adjusted their cooperation with individuals whose cooperation patterns deviate from the group cooperation tendency. Overall, all participants cooperated more with individually cooperative partners, and less with individually noncooperative partners. However, although both older and younger participants learned to discriminate between cooperative and noncooperative partners, older participants took less benefit from their experience with their partners than younger participants in the final part of the experiment. The fact that this impaired learning was only observed at the end of the experiment and was selective to inconsistent partners suggests that older partners’ attentional resources may have started to be depleted, primarily affecting controlled processes, but not automatic responses based on categorical information (Govorun and Payne, 2006).

Despite these differences, older participants’ performance was overall quite similar to their younger counterparts, and the differences observed between the two age groups were subtle. Although we hypothesized that older adults would be more impacted by categorical cues than younger adults because of the resources-saving function of social categorization, it is worth considering that beyond cognitive cost, motivational factors may have played a role in promoting the use of individuating information in the Trust Game. In fact, research suggests that older adults are more motivated to achieve harmonious relationships (Carstensen et al., 1999; Luong et al., 2011) which may have been a key factor contributing to their performance. Furthermore, motivation to individuate may be enhanced by providing participants economic rewards according to their accuracy in a task (e.g., Kawakami et al., 2014). The fact that in this experiment, participants were financially rewarded may have enhanced their motivation to respond accurately, promoting the engagement of the cognitive resources necessary to overcome social biases and individuate their partners.

Importantly, these results echo previous research suggesting that although facial cues may impact our first impressions of others, these superficial judgments tend to be overridden when we are presented with relevant behavioral information (Chang et al., 2010; Shen et al., 2020). For instance, Rezlescu et al. (2012) presented participants with game partners whose facial features were manipulated to be associated with either high or low trustworthiness. In a series of two experiments, they observed that these facial features largely impact cooperation decisions as participants tended to cooperate more with partners whose faces appeared more trustworthy. However, when information about how these partners behaved in the past was made available to participants, this reputational information was primarily used and the impact of facial features on cooperation drastically reduced (i.e., from 42% to 6%). Similar results were found when analyzing the impact of facial affective cues on cooperation. For example, Campellone and Kring (2013) found that when facial emotional expressions and individual behaviors provide incongruent information (e.g., a person who tends to cooperate appears with an angry face), participants primarily rely on the behavioral information to make their cooperation decision. Overall, it seems that the impact of facial features on cooperation decisions progressively decays as the participant accumulates behavioral information related to their partners’ cooperation tendency (Chang et al., 2010; Bailey et al., 2019).

Although the impact of age-related categorical information was fairly limited, we did observe, that in first interactions with partners, older participants consistently relied on gender stereotypes and cooperated more with female than with male partners (Brañas-Garza et al., 2018; Telga et al., 2018), while this effect was less consistent among younger participants. These data provide evidence for the hypothesis that in first interactions, older adults may be more likely to rely on social heuristics than younger adults, although in the present research, participants primarily used gender (as opposed to age) dimension for social categorization. They also suggest that in first encounters, age categories did not trigger the same categorization processes as gender in the present research, or gender and ethnicity in previous research. For instance, using the exact same paradigm, Telga et al. (2018) observed that ethnic categories (i.e., black and white partners) triggered an outgroup favoritism, while gender categories (i.e., male vs. female partners) were associated with an ingroup favoritism. Importantly, these experiments used the same sample size as used in the current research (and therefore, were equally powered to detect the effects of interest), suggesting that in a Trust Game, if any, age-based categorization effects at zero acquaintance are of a smaller size than the ones observed for gender or ethnicity.

Although the present research primarily focused on age social categories, the reliance on gender categories in this context is not surprising. Women are typically perceived to have more communal traits than men (Cuddy et al., 2008, 2009). Consequently, in trust settings, people often cooperate more with female than with male partners (Buchan et al., 2008; Brañas-Garza et al., 2018; Telga et al., 2018). The absence of reliance on age stereotypes in first encounters, however, was not expected. Research in social psychology has largely demonstrated that age is central in impression formation (Montepare and Zebrowitz, 1998) and that categorical judgments based on age are equally activated by younger and older adults (Bassili and Reil, 1981; Chasteen et al., 2002). Moreover, there is evidence suggesting that when it comes to stereotyping, age may outweigh other social categories (Bassili and Reil, 1981; Kite et al., 1991). Following the Stereotype Content Model (Fiske et al., 2002), we expected older adults to be perceived higher on the warmth dimension than younger adults (Cuddy et al., 2008), and therefore to be trusted to a greater extent. However, the stereotype of older adults is multidimensional and beyond warmth, includes traits, such as conservative, traditional, present-oriented, moral (Bassili and Reil, 1981), and overall less competent and agentic (Kite et al., 1991; Cuddy et al., 2005). Because these characteristics are not really related to the likelihood that a person cooperates in a Trust Game, age categories may have been perceived as less relevant than gender, leading participants to focus on the latter. This interpretation is consistent with intersectional and dynamic theories of person construal (Quinn and Macrae, 2005; Freeman and Ambady, 2011; Remedios and Sanchez, 2018) suggesting certain contexts may enhance the salience of a specific social dimension, making more likely to categorize a target based on the salient category. In other words, it is possible that the task emphasizing trust and interpersonal relationships has made gender more salient than age and that although participants activated age-related stereotypes, these were disregarded to the extent they were considered irrelevant in the context of the trust game.

Furthermore, a unique aspect of age as a social dimension for categorization is that everybody *necessarily* goes through all age categories across the life span, and belonging to a particular age group is only temporal. This may contribute to the perception that the frontiers between age groups are particularly malleable, and therefore, reduce the salience of age in certain contexts. Additionally, when it comes to the social dimension of age, higher status and benefits at the economic and social levels are typically enjoyed by middle-aged people (Fiske, 2010). In the present experiment, older and younger participants fell outside this privileged class, which may have enhanced a shared identity of low status related to age, potentially decreasing the expected intergroup processes. Although age, gender, and ethnicity are often considered the “big three” of social categorization (Stolier and Freeman, 2016), further research is needed to better understand the differences between these three dimensions and why they may trigger different categorization processes.

### Limitations and Future Directions

We acknowledge several limitations of the present research. First, it is unclear whether the absence of age-based stereotypes is the result of a lack of relevance of such stereotypes in the Trust Game (i.e., participants activated but did not use the stereotype in this context), or a lack of endorsement of age stereotypes by the participants taking part in this study. Future research may help to disambiguate these processes by assessing the endorsement of age stereotypes in a separate task. Second, more research is needed to better understand whether the impaired performance of older partners at the end of the experiment is the result of a lack of flexibility to relearn new associations between groups and cooperative behaviors, cognitive fatigue, vigilance decrement, or working memory deficit. Additional measures of cognitive control, cognitive flexibility, vigilance, and working memory may shed led light on the specific mechanisms at stake in the last stage of the Trust Game. Finally, further investigation is needed to understand the boundaries and generalizability of the present results. In particular, the sample of older participants used in the current research showed very high scores in the MMSE and was generally still employed. Employment, cognitive stimulation, or socioeconomic status may potentially account for the remarkable performance of older participants in the present experiment and should be taken into account in future research. Despite its limitation, we believe that this research provides relevant evidence of the differential use of categorical and individuating information by older and younger adults in several instances, including first encounters, learning across repeated interactions, and member-to-member generalization.

### Conclusion

Overall, the data from this study suggest that older adults may rely more on social heuristics than younger adults, but this difference is limited to first impressions and may be overridden with experience. The information allowing people to individuate others may be progressively prioritized over easily noticeable categorical cues by both older and younger adults. Even in a context where categorical information is relevant, people may ignore it in favor of behavioral cues offering more accuracy to inform their judgments. Importantly, they also suggest that as well as cognitive resources may constrain social perception, enhanced motivation may help older adults to reduce biases in intergroup contexts.

## Data Availability Statement

The dataset analyzed for this study can be found on Open Science Framework: https://osf.io/sx3jw/?view_only=6ab6367677184f9888e2960624e3fa16.

## Ethics Statement

The studies involving human participants were reviewed and approved by the University of Granada (175/CEIH/2017). The patients/participants provided their written informed consent to participate in this study.

## Author Contributions

MT and JL conceived and designed the studies and interpreted the data. MT programmed the experiments, collected and analyzed the data, and wrote the manuscript which was critically revised by JL. All authors contributed to the article and approved the submitted version.

## Funding

This work was supported by the Spanish Ministry of Education, Culture and Sports, with pre-doctoral FPU fellowship FPU14/07106 to MT, and the Spanish Ministry of Economy and Competitiveness, with research projects PSI2014-52764-P and PSI2017-84926-P to JL. This research is part of MT’s thesis dissertation under the supervision of JL.

## Conflict of Interest

The authors declare that the research was conducted in the absence of any commercial or financial relationships that could be construed as a potential conflict of interest.

## Publisher’s Note

All claims expressed in this article are solely those of the authors and do not necessarily represent those of their affiliated organizations, or those of the publisher, the editors and the reviewers. Any product that may be evaluated in this article, or claim that may be made by its manufacturer, is not guaranteed or endorsed by the publisher.

## Footnotes

^1^ https://osf.io/sx3jw/?view_only=6ab6367677184f9888e2960624e3fa16

^2^Specifically, we combined the first block of the first learning phase (Block 2) with the first block of the second learning phase (Block 7), the second block of the first learning phase (Block 3) with the second block of the second learning phase (Block 7), the third block of the first learning phase (Block 4) with the third block of the second learning phase (Block 9), and the fourth block of the first learning phase (Block 5) with the fourth block of the second learning phase (Block 10) and labeled the combination of blocks as Block II (Blocks 2 and 7), Block III (Blocks 3 and 8), Block IV (Blocks 4 and 9), and Block V (Blocks 5 and 10).
