# Supplementary material for: Older and Younger Adults Perform Similarly in an Iterated Trust Game
Source: Front Psychol. 2021 Oct 12;12:747187. doi: 10.3389/fpsyg.2021.747187 (PMC8547485; doi:10.3389/fpsyg.2021.747187)
Supplement: Supplementary file 1 [file Data_Sheet_1.docx]

Supplementary Material

# Supplementary Data

## First Learning Phase: Blocks 2 to 5

Cooperation rates in Blocks 2 to 5 were subjected to a mixed-design ANOVA with participant age (older vs younger adults) and cooperative group (old vs young partners) as between-participants factors, and group behavior (cooperative vs noncooperative), consistency (consistent vs inconsistent) and blocks (2, 3, 4, 5) as within-participants variables.

The expected Group Behavior x Consistency x Block interaction was significant, *F*(3, 231) = 26.88, *p* < .001, $ƞ_{p}^{2}$ = .26, 95% CI [ .18, .33]. We decomposed this interaction by first analyzing cooperation decisions with consistent partners before turning to cooperation with inconsistent partners.

In the consistent partners condition, the Group Behavior x Block was significant, *F*(3, 240) = 33.44, *p* < .001, $ƞ_{p}^{2}$ **=** .30, 95% CI = [.21, .36]. In fact, participants linearly increased their cooperation with cooperative partners from Block 2 (*M* = .66, *SD* = .19) to Block 5 (*M* = .76, *SD* = .23), *F*(1, 80) = 16.01, *p* < .001, $ƞ_{p}^{2}$ **=** .17, 95% CI = [.06, .28]. In contrast, participants linearly decreased their cooperation with noncooperative partners from Block 2 (*M* = .60, *SD* = .20) to Block 5 (*M* = .47, *SD* = .25), *F*(1, 80) = 28.61, *p* < .001, $ƞ_{p}^{2}$ = .26, 95% CI = [.13, .38]. The quadratic component was also significant, *F*(1, 80) = 9.21, *p* = .003, $ƞ_{p}^{2}$ = .10, 95% CI = [.02, .21], indicating that this decrease reached an asymptote.

In the inconsistent partners condition, when partners displayed cooperative behaviors opposite to the group behavior, the Group Behavior x Block interaction was also significant, *F*(3, 240) = 8.65, *p* < .001, $ƞ_{p}^{2}$ **=** .10, 95% CI = [.04, .15] showing that participants accurately adjusted their cooperation with inconsistent partners. Specifically, they tended to increase their cooperation with inconsistent partners belonging to the noncooperative group from Block 2 (*M* = .70, *SD* = .29) to Block 5 (*M* = .77, *SD* = .26), *F*(1, 80) = 3.93, *p* = .051, $ƞ_{p}^{2}$ = .05, 95% CI = [.00, .14], and linearly decreased their cooperation with inconsistent partners belonging to an cooperative group from Block 2 (*M* = .65, *SD* = .26) to Block 5 (*M* = .52, *SD* = .33), *F*(1, 80) = 13.00, *p* = .001, $ƞ_{p}^{2}$ = .14, 95% CI = [.05, .26], until reaching an asymptote, quadratic component, *F*(1, 80) = 8.32, *p* = .005, $ƞ_{p}^{2}$ **=** .09, 95% CI = [.02, .20].

Overall, both older and younger participants accurately displayed opposite patterns of cooperation with consistent and inconsistent partners from the same age group, suggesting that when deciding whether or not to cooperate with their partners, all participants adopted an individuating approach. The main effect of Participant Age was not significant, *F*(1, 77) = 0.20, *p* = .654, $ƞ_{p}^{2}$ < .01, 95% CI [ .00, .05] indicating that Hypothesis 2d was not supported.

## Second Learning Phase: Block 7 to 10

Cooperation rates in Blocks 7 to 10 were subjected to a mixed-design ANOVA with participant age (older vs younger adults) and cooperative group (old vs young partners) as between-participants factors, and group behavior (cooperative vs noncooperative), consistency (consistent vs inconsistent) and blocks (7, 8, 9, 10) as within-participants variables.

Again, the expected Group Behavior x Consistency x Block interaction was significant, *F*(3, 231) = 14.70, *p* < .001, $ƞ_{p}^{2}$ = .16, 95% CI [ .09, .22]. The four-way Participant Age x Group Behavior x Consistency x Block interaction was not significant but close, *F*(3, 231) = 2.48, *p* = .062, $ƞ_{p}^{2}$ = .03, 95% CI [ .00, .07]. Because this interaction was directly related to Hypothesis 2c, we decided to decompose it by first examining whether older and younger participants differ when responding to consistent partners, and next whether older and younger participants differ when responding to inconsistent partners.

In the consistent partners condition, the Group Behavior x Block interaction was significant, *F*(3, 237) = 16.61, *p* < .001, $ƞ_{p}^{2}$ = .17, 95% CI = [.10, .24], and not qualified by Participant Age, *F*(3, 237) = 0.29, *p* = .833, $ƞ_{p}^{2}$ < .01, 95% CI = [.00, .01], suggesting that older and younger adults learned to similar extent the cooperation tendencies of consistent partners. In fact, both older and younger participants linearly increased their cooperation with cooperative partners from Block 7 (*M* = .69, *SD* = .23) to Block 10 (*M* = .74, *SD* = 24), *F*(1, 80) = 8.74, *p* = .004, $ƞ_{p}^{2}$ = .10, 95% CI = [.02, .21] until reaching an asymptote, quadratic component, *F*(1, 80) = 6.12, *p* = .016, $ƞ_{p}^{2}$ = .07, 95% CI = [.01, .17], and linearly decreased their cooperation with noncooperative partners from Block 7 (*M* = .60, *SD* = .23) to Block 10 (*M* = .50, *SD* = .27), *F*(1, 80) = 22.54, *p* < .001, $ƞ_{p}^{2}$ = .22, 95% CI = [.10, .34].

In the inconsistent partners condition, however, the Group Behavior x Block was significant, *F*(3, 237) = 5.56, *p* = .001, $ƞ_{p}^{2}$ = .07, 95% CI = [.02, .11], and qualified by Participant Age, *F*(3, 237) = 5.13, *p* < .002, $ƞ_{p}^{2}$ = .06, 95% CI = [.01, .11], indicating that older and younger participants differed in the way they responded to inconsistent partners.

Specifically, and as observed in the first learning phase, younger participants accurately increased their cooperation with inconsistent partners belonging to the noncooperative group from Block 7 (*M* = .71, *SD* = .26) to Block 10 (*M* = .81, *SD* = .28), *F*(1, 40) = 4.88, *p* = .033, $ƞ_{p}^{2}$ = .11, 95% CI = [.01, .26], and decreased their cooperation with inconsistent partners belonging to the cooperative group from Block 7 (*M* = .66, *SD* = .28) to Block 10 (*M* = .45, *SD* = .34), *F*(1, 40) = 17.76, *p* < .001, $ƞ_{p}^{2}$ = .31, 95% CI = [.12, .46].

However, older participants did not increase their cooperation with inconsistent partners belonging to the noncooperative group from Block 7 (*M* = .73, *SD* = 26) to Block 10 (*M* = .71, *SD* = .31), *F*(1, 39) = 0.41, *p* = .526, $ƞ_{p}^{2}$ = .01, 95% CI = [.00, .01]. Neither did they decrease their cooperation with inconsistent partners belonging to the cooperative group from Block 7 (*M* = .62, *SD* = .27) to Block 10 (*M* = .59, *SD* = .31), *F*(1, 39) = 0.99, *p* = .327, $ƞ_{p}^{2}$ = .03, 95% CI = [.00, .15], suggesting that the general tendency to cooperate more with individually cooperative individuals and less with individually noncooperative individuals did not increase across blocks.

Overall, younger participants accurately displayed opposite patterns of cooperation with consistent and inconsistent partners from the same age group, and this tendency increases across blocks of trials. Although older participants also cooperated more with individually cooperative inconsistent (M = .71, SD = .60) partners and less with individually noncooperative inconsistent partners (M = .60, SD = .26), *F*(1, 39) = 8.18, *p* = .007, $ƞ_{p}^{2}$ = .17, 95% CI = [.03, .34], this tendency did not increase as the second learning phase progressed. The main effect of Participant Age was not significant, *F*(1, 77) = 0.15, *p* = .696, $ƞ_{p}^{2}$ < .01, 95% CI [ .00, .05] indicating that Hypothesis 2d anticipating that older participants are more cooperative than younger participants was not supported.
